# Nano-Chitosan/*Eucalyptus* Oil/Cellulose Acetate Nanofibers: Manufacturing, Antibacterial and Wound Healing Activities

**DOI:** 10.3390/membranes13060604

**Published:** 2023-06-15

**Authors:** Nagwa A. Elbhnsawi, Bassma H. Elwakil, Ahmed H. Hassanin, Nader Shehata, Salma Sameh Elshewemi, Mohamed Hagar, Zakia A. Olama

**Affiliations:** 1 Department of Botany & Microbiology, Faculty of Science, Alexandria University, Alexandria 21568, Egypt; 2 Department of Medical Laboratory Technology, Faculty of Applied Health Sciences Technology, Pharos University in Alexandria, Alexandria 21500, Egypt; 3 Centre of Smart Materials, Nanotechnology and Photonics (CSNP), SmartCI Research Centre, Alexandria University, Alexandria 21544, Egypt; 4 Department of Textile Engineering, Faculty of Engineering, Alexandria University, Alexandria 21544, Egypt; 5 Wilson College of Textiles, North Carolina State University, Raleigh, NC 27695, USA; 6 Department of Engineering Mathematics and Physics, Faculty of Engineering, Alexandria University, Alexandria 21544, Egypt; 7 USTAR Bio Innovations Centre, Faculty of Science, Utah State University, Logan, UT 84341, USA; 8 Department of Physics, School of Engineering, Kuwait College of Science and Technology (KCST), Doha Superior Rd., Jahraa 13133, Kuwait; 9 Department of Zoology, Faculty of Science, Alexandria University, Alexandria 21568, Egypt; 10 Department of Chemistry, Faculty of Science, Alexandria University, Alexandria 21568, Egypt

**Keywords:** wound dressing nanofibers, antibacterial effect, inflammation, wound healing

## Abstract

Accelerated wound healing in infected skin is still one of the areas where current therapeutic tactics fall short, which highlights the critical necessity for the exploration of new therapeutic approaches. The present study aimed to encapsulate *Eucalyptus* oil in a nano-drug carrier to enhance its antimicrobial activity. Furthermore, in vitro, and in vivo wound healing studies of the novel nano-chitosan/*Eucalyptus* oil/cellulose acetate electrospun nanofibers were investigated. *Eucalyptus* oil showed a potent antimicrobial activity against the tested pathogens and the highest inhibition zone diameter, MIC, and MBC (15.3 mm, 16.0 μg/mL, and 256 μg/mL, respectively) were recorded against *Staphylococcus aureus*. Data indicated a three-fold increase in the antimicrobial activity of *Eucalyptus* oil encapsulated chitosan nanoparticle (43 mm inhibition zone diameter against *S. aureus*). The biosynthesized nanoparticles had a 48.26 nm particle size, 19.0 mV zeta potential, and 0.45 PDI. Electrospinning of nano-chitosan/*Eucalyptus* oil/cellulose acetate nanofibers was conducted, and the physico-chemical and biological properties revealed that the synthesized nanofibers were homogenous, with a thin diameter (98.0 nm) and a significantly high antimicrobial activity. The in vitro cytotoxic effect in a human normal melanocyte cell line (HFB4) proved an 80% cell viability using 1.5 mg/mL of nano-chitosan/*Eucalyptus* oil/cellulose acetate nanofibers. In vitro and in vivo wound healing studies revealed that nano-chitosan/*Eucalyptus* oil/cellulose acetate nanofibers were safe and efficiently enhanced the wound-healing process through enhancing TGF-β, type I and type III collagen production. As a conclusion, the manufactured nano-chitosan/*Eucalyptus* oil/cellulose acetate nanofiber showed effective potentiality for its use as a wound healing dressing.

## 1. Introduction

The body’s ability to repair injured tissue via the healing process is a miracle of nature. The wound healing process includes hemostasis, inflammation, proliferation, remodeling and scar formation [1], which takes a very long time with the possibility of pathogenic microbes to contaminate the wounded area. Thus, immediate external help is required to enhance and accelerate this process [2]. Infectious and inflammatory conditions, immune system strength, and blood circulation efficacy may all interfere with the wound healing process [3]. A limited number of antibiotics are currently used in wound healing applications due to their limited cell uptake and high toxicity [4]. When microorganisms become resistant to antibiotics, the efficiency of antibiotic therapy decreases, and potentially increases the morbidity and mortality incidences. Antibiotics that were formerly very successful in the combat against pathogenic bacteria now seem to have the opposite effect. Among the Gram-positive bacteria, methicillin-resistant *Staphylococcus aureus* (MRSA) is the most concerning resistant pathogen. A study in Australia found that two-thirds of diabetic foot ulcer patients were infected with *S. aureus*, and nearly half were methicillin-resistant (MRSA), for which there are limited antimicrobial treatment options [5]. Hence, antibiotic resistance is a major obstacle in treating infections caused by this pathogen [6]. For this reason, it is critical to rapidly identify and develop novel antimicrobial medicines against multidrug-resistant pathogenic bacteria [7].

Even though wound healing may be slowed in certain cases, several options exist, from non-invasive wound care management to invasive surgical procedures. Wound therapies have progressed beyond ointments and dressings only to polyurethanes, hyaluronic acid, and synthetic growth factor hydrogels [8]. Newer therapies were invented to provide a moist environment that promotes recovery. Inconveniently, most of the current therapies have their drawbacks, such as high costs, the evolution of microbial resistance, and allergic responses. Alternatives provided by natural resources are thought to have little harmful consequences [9].

Natural therapies with potent healing effects have been more popular in recent years for treating a wide range of skin problems with improved safety standards [10]. The genus *Eucalyptus* includes several species of flowering trees and shrubs from the Myrtaceae family. *Eucalyptus* essential oils may be used in several biomedical applications, including as an anti-inflammatory, antispasmodic, decongestant, deodorant, antiseptic, and antibacterial agents. Cineol, one of *Eucalyptus* essential oil chemical constituents, has been linked to the reported antibacterial properties. Of the 800 species of *Eucalyptus*, only around 20 species can produce an essential oil that particularly has high concentration of cineole [11]. These include species such as *Eucalyptus globulus*, *E. camaldulensis*, *E. tereticornis*, and *E. citriodora*. Essential oils have great potential as pharmacotherapeutic treatment options, but their use is limited due to several problems. Because of the risk of an allergic response, direct contact with the skin is discouraged. If these volatile non-stable essential oils are not protected from oxidation, evaporation, heat, and light, they will quickly disintegrate [12]. Nano-encapsulation technology, a contemporary medication delivery technique, is one approach to overcome these limitations [2]. Moreover, because of their plentiful supply, high biocompatibility, high chemical/physical properties, degradability, low cost, large-scale production, and versatile use for composite materials, cellulose nanomaterials such as cellulose acetate nanofibers have received considerable attention as a next-generation nanomaterial [13]. Nanomaterials derived from cellulose have several desirable qualities [14]. Cellulose acetate nanomaterials can be used in food packaging, wound dressings, sensors, energy storage systems, water purification, and as industrial materials [15]. Nanofiber-based products have been used in several fields, namely public health, air/water filtration, energy storage, etc. After the COVID-19 pandemic, the demand for nonwoven products rapidly increased [16]. Electrospinning is the most popular technology with which to produce nanofiber-based products from various kinds of materials on bench and commercial scales [17].

Hence, the present investigation aimed to assess the potential use of the prepared *Eucalyptus* oil/cellulose acetate and nano-chitosan/*Eucalyptus* oil/cellulose acetate nanofibers as efficient wound dressings to inhibit bacterial wound infections and promote the wound healing process. To the best of our knowledge, this is the first work to assess the antimicrobial and wound healing efficiencies of newly designed nano-chitosan/*Eucalyptus* oil/cellulose acetate nanofibers with special emphasis on the underlying mechanisms.

## 2. Materials and Methods

### 2.1. Materials

Cellulose acetate (MW ∼100,000 Da; acetyl content ∼39.7 wt%) was purchased from VWR International (VWR international, Radnor, PA, USA). Low-molecular-weight chitosan (100–150 kDA, DDA ≈ 85%) and sodium tripolyphosphate (TPP) were purchased from Thermo Fisher Scientific (Thermo Fisher Scientific Inc., Waltham, MA, USA). The enzyme-linked immunosorbent assay (ELISA) kits used throughout the present work were purchased from Shanghai X-Y Biotechnology Co.

### 2.2. Microorganisms

All the bacterial strains used throughout the present work, namely Klebsiella pneumonia, Acinetobacter baumanii, Escherichia coli, Proteus Vulgaris, Pseudomonas aeruginosa, Candida albicans, and Staphylococcus aureus, were kindly provided by the Surveillance Microbiology Department’s strain bank at the main University Hospital, Alexandria University in Alexandria, Egypt, and further identification was performed by using the Vitek 2 automated system (bioMerieux, Marcy l’Etoile, France) at the Medical Research center, Faculty of Medicine, Alexandria.

### 2.3. Hydrodistillation Extraction and Chemical Analyses

*Eucalyptus camaldulensis* leaves (150 g of fresh leaves) were used in the hydrodistillation process using a Clevenger apparatus for 4 h as described in the European Pharmacopoeia. The extracted EO was stored in brown glass vials at 4 °C for further analyses [18].

#### Gas Chromatography–Mass Spectroscopy (GC-MS) and FTIR Analyses

GC-MS analysis (Perkin Elmer Instruments, Norwalk, CT 06859, USA) equipped with Rtx-5 Capillary Column (Resstek, Bellefonte, PA, USA; 0.25 mm diameter, with Crossbond 5% diphenyl-95% -dimethyl polysiloxane). The mass detector operated in the electronic ionization mode (EI+) at a scan rate of 0.10 s^−1^ and a mass range of 40–400. The extracted oil sample was diluted with n-hexane (3 mg/mL) and then injected into the autosystem. Individual peak identities were predicted by comparing mass spectral data to a National Institute of Standards and Technologies (NIST) mass spectral library reference [19].

FTIR analysis (Perkin-Elmer R79521, Buckinghamshire, UK; 2 cm^−1^ resolution; wave number ranged between 4000 and 450 cm^−1^ during 64 scans) was performed to detect the functional groups present in the extracted oil sample [20,21].

### 2.4. Antimicrobial Activity

The antibacterial activity of the extracted oil was investigated using the disc diffusion method, and minimum inhibitory concentration (MIC), minimum bactericidal concentration (MBC) and MIC index assessments using the microdilution technique in accordance with Diab et al. [22] and EUCAST [23]. The minimal inhibitory concentration was the lowest essential oil concentration at which no visible growth was detected, while the MBC was recorded when 99.9% of the bacterial population was killed at the lowest concentration of the extracted essential oil. The MIC index is the MBC/MIC ratio, and is used to assess antibacterial mechanistic action. When the MIC index was equal to 1 or 2, the effect was considered bactericidal while if the MIC index was equal to 4 or more, the effect was defined as bacteriostatic [23].

### 2.5. Nano-Eucalyptus Oil Synthesis

#### 2.5.1. Nano-Chitosan/*Eucalyptus* Oil

Chitosan nanoparticles encapsulated with *Eucalyptus* oil were prepared according to Ribeiro et al. [24]. A mixture of EO and Tween 80 at a ratio of 2:1 was added to a solution of 2% chitosan (*v*/*v*) and sodium TPP (0.2% *w*/*v* in deionized water) with stirring at 50 rpm for 10 min. The suspension obtained was subjected to ultracentrifugation at 25,000 rpm for 20 min and the precipitate was stored at 4 °C in sterile falcon tubes for further investigations.

#### 2.5.2. Antimicrobial Activity of the Prepared Nano-Chitosan/*Eucalyptus* Oil

The antibacterial activity of the prepared nano-chitosan/oil (25 mg/mL) nanofiber was investigated using the disc diffusion method, MIC and MBC according to Diab et al. [22]. Nano-chitosan/*Eucalyptus* oil showed the highest antimicrobial activity and was selected for further analyses.

#### 2.5.3. Physico-Chemical Characterization of the Most Promising Nano-Chitosan/*Eucalyptus* Oil Nanoparticles

The particle size (PS), polydispersity index (PDI), and Zeta potential of the most promising nano-chitosan/*Eucalyptus* oil were determined using a dynamic light scattering (DLS) technique (Malvern Zetasizer, Worcestershire, UK). The ultra-structure, size, and shape of the prepared nano-chitosan/*Eucalyptus* oil nanofiber were examined via transmission electron microscopy (TEM) [25].

### 2.6. Manufacturing of Nanofibrous Membranes

Briefly, 10 g of cellulose acetate (CA) was dissolved in acetone/Dmac (2/1) with continuous stirring at room temperature. Crude *Eucalyptus* oil and nano-chitosan/*Eucalyptus* oil (15% each) were blended with the prepared cellulose acetate solution one at a time with continuous stirring (300 rpm, at room temperature for 3 h). The electrospinning of the prepared solutions was performed using a plastic syringe with an 18-gauge stainless steel needle while a high voltage power supply (25 kV) (CZE1000R Spellman, Hauppauge, NY, USA) was applied with a flow rate of 1 mL/h. The nanofibrous membranes were collected on the collector with a 17 cm diameter, and 20 cm was the distance between the needle and collector [26].

#### 2.6.1. Morphological and Physical Characterizations of the Prepared Electrospun Nanofibers

The morphology of the electrospun nanofibers was investigated using scanning electron microscopy (SEM) (JEOL, JSM-6010LV-SEM, Tokyo, Japan). The average fiber diameter and distribution were measured using software (Madison, WI, USA), while FTIR spectroscopy (Perkin Elmer, Inc., Waltham, MA, USA) was conducted with a 2 cm^−1^ resolution and wavenumber of 4000 cm^−1^ to 450 cm^−1^ in sixty-four scans to determine the characteristic functional groups of the electrospun nanofibers [16]. X-Ray diffraction (XRD) patterns were measured to evaluate the existence of the fabricated nano-chitosan/*Eucalyptus* oil nanoparticles in the core layer using an X-ray powder diffractometer (XRD- BRUKER AXS) with a Ni filter, and a Cu Kα radiation source (λ = 0.154 nm), at a scan rate of 10°/min, using a voltage of 40 kV and a current of 30 mA [27].

On the other hand, the Micro 50 fiber strength tester from Shirley Co. was used to measure the durability of the manufactured nanofibers. The five measurements taken at 5 mm/min and a gauge length of 10 mm for each sample were averaged for reporting [12].

Moreover, the release behavior of nano-chitosan/*Eucalyptus* oil and *Eucalyptus* oil nanofibers from the prepared nano-chitosan/*Eucalyptus* oil/cellulose acetate nanofibers was evaluated in PBS for 24 h in accordance with Ye et al. [28].

#### 2.6.2. Loading Analysis

Using UV spectroscopy at a wavelength of 390 nm, the loading efficiency (LE%) of the nanofibers was determined by dissolving a sample with a known mass of the produced nanofibers in acetone/Dmac at room temperature (Equation (1)) [29]:

(1) $$LE\%=\frac{Initial\;drug\;concentration\;-\;Loaded\;drug\;concentration}{Initial\;drug\;concetration}\times 100$$

### 2.7. In Vitro Studies

#### 2.7.1. Antibacterial Activity of the Prepared Electrospun Nanofibers

According to ASTME 214901 standards [30] (Standard Test Method for Determining the Antimicrobial Activity of Immobilized Antimicrobial Agents Under Dynamic Contact Conditions), the antimicrobial activity of the produced nanofibers was estimated by calculating the percent decrease in each test organism after incubation with the nanofibers for 24 h using the following formula (Equation (2)):

(2) $$R\%=\frac{B-A}{B}\times 100$$

R% is the reduction percentage of the colony-forming unit, A is the number of colony forming units per flask that was supplemented with the prepared nanofibers after 24 h of incubation, and B is the initial number of the colony forming unit per flask before the nanofiber’s addition [31]. Moreover, the antimicrobial activity was further assessed by agar diffusion method [16].

##### Fluorescence Microscopy (Live/Dead Cell Assay)

The antibacterial effect of the most potent nanofibers was studied using confocal scanning laser microscopy (CLSM) with a Leica DMI 6000 B FluoView microscope (TCS SP5) coupled to a confocal scanner (USA). Nanofiber samples with standard dimensions (1 cm × 1 cm) were added to bacterial consortium suspension (*P. vulgaris*/*C. albicans*/*S. aureus*: 1/1/1 *v*/*v* of 10^2^ CFU/mL). Confocal scanning laser microscopy was provided by a Kr/Ar laser (488 nm laser excitation) fitted with a long-pass 514 nm emission filter. A 580 nm beam splitter was used together with a long-pass 520 nm filter (green fluorescence signal) and long-pass 590 nm filter (red fluorescence signal). Simultaneous dual-channel imaging using pseudocolor was used to display green and red fluorescence [32].

##### Scanning Electron Microscopy (SEM)

A SEM study was conducted by adding the nanofiber samples with standard dimensions (1 cm × 1 cm) to a Staphylococcus aureus suspension (10^2^ CFU/mL). After 24 h of incubation, the nanofibers were collected and examined under a scanning electron microscope [26].

#### 2.7.2. Evaluation of the Cytotoxic Effect of the Prepared Electrospun Nanofibers

In the present study, HFB4 cells were used (a human normal melanocyte cell line from the American Type Culture Collection (ATCC, Rockville, MD, USA)). The melanocyte cell line was chosen due to the reported specific cytotoxic effect of some phenolic compounds (major constituents in essential oils) on melanocytes [33,34,35]. Inactivated fetal calf serum (A 10%) and 50 μg/mL gentamycin were added to RPMI 1640 medium for cell cultivation. The cells were subcultured twice weekly at 37 °C in a humidified environment containing 5% carbon dioxide. The cell lines were seeded at a density of 5 × 10^4^ cells/well in Corning^®^ 96-well tissue culture plates, then incubated for 24 h. Fixed nanofiber weights were diluted to eight different concentrations before being inoculated in 96-well plates (in three duplicates). Cell viability was assessed using the MTT (3-(4,5-dimethylthiazol-2-yl)-2,5-diphenyltetrazolium bromide) assay after 24 h of incubation. The main principle of MTT assay is the reduction of tetrazolium salt by mitochondrial succinic dehydrogenases (present in viable cells) which results in MTT-formazan insoluble crystals (from a blue to purple color). MTT-formazan crystals were then dissolved using 20% SDS and 50% DMF, at pH 4.7 with overnight incubation, and then the absorbance was measured at 570 nm. For each cell line, the survival viability was calculated by plotting the percentage of cells that survived against the different concentrations of the tested nanofibers. Using GraphPad Prism 5 (San Diego, CA, USA), dose–response curves were plotted for each concentration to determine the cytotoxic concentration (CC50) (the dosage needed to elicit lethal effects in 50% of the intact cells) [26,36].

#### 2.7.3. In Vitro Scratch Assay of the Prepared Nanofibers

In 24-well PermanoxTM plates, human epithelial cells were cultured until they reached confluence. A sterile 10 mL pipette tip was used to make a uniform wound across each well, separating the cells. In 1% FBS-supplemented culture media, the cells were then exposed to the prepared nanofibers. Similarly, control cells were scratched, washed, and cultured in a medium containing 1% FBS. Phase-contrast microscopy photographs of the scraped regions were taken immediately after the scratch (zero time) then after 24 h. Six spots on each picture were used to measure the scratch breadth and the remaining injured area [37].

### 2.8. In Vivo Study

#### 2.8.1. Animal Modeling

**Scheme 1:**
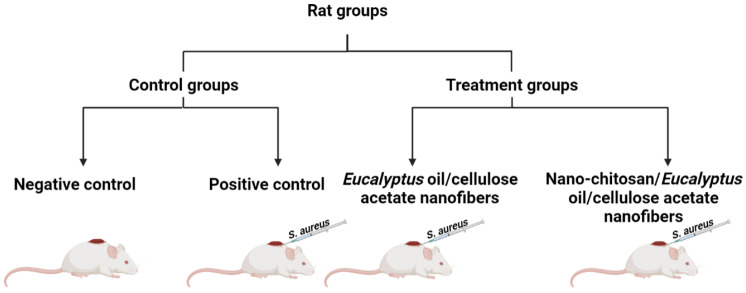
Schematic representation of assigned rat groups.

Four-month-old, male albino rats (*Rattus norvegicus Albinus*) weighing 180 ± 30 g were adapted (1 week); all animal experiments were conducted according to the ethics approval that was obtained from the Care and Use of Laboratory Animals approved by the Institutional Animal Care and Use Committees (IACUCs) of the Faculty of Science, Alexandria University, and were conducted in accordance with the International Standards for the Care and Use of Laboratory Animals of the European Community Directive of 1986, AU 04/23/04/27/1/03. After adaption, rats were split into four groups. All the rat groups (ten rat/group) were housed in a well-ventilated room at a constant temperature and humidity of 25 ± 2 °C for 30 days. The dorsal hair of each rat was shaved off, followed by making an artificial wound measuring 1 cm^2^ in diameter, then 50 μL of *S. aureus* (1.0 × 10^7^ CFU/mL) was intradermally injected. All rats were kept in a standard 12 h light/12 h dark cycle with unrestricted access to food and water, and were then divided and separated into four groups (Scheme 1) according to the following treatment protocols:

- Group I: assigned as a negative control (normal, neither infected nor treated).
- Group II: assigned as a positive control (infected with *S. aureus* with no treatment).
- Group III: *S. aureus*-infected rats treated with *Eucalyptus* oil/cellulose acetate nanofibers.
- Group IV: *S. aureus*-infected rats treated with nano-chitosan/*Eucalyptus* oil/cellulose acetate nanofibers.

The rat groups were treated daily for 7 days with 1 cm × 1 cm nanofibers one at a time (applied directly to the wound site as a wound dressing to cover the wound in the treated groups and changed daily). The progressive changes in the wound area were monitored, and the wound area was measured every day. The wound size on day 0 was set to 100%, and that on each subsequent day was reported as a percentage of the initial wound size. The percentage of wound contraction was calculated with the following formula (Equation (3)):

(3) $$WC=\frac{S_{0}-S}{S_{0}}\times 100$$

where S_0_ is the initial wound area and S is the wound area on a specific day [21].

#### 2.8.2. Bacterial Load Assessment

**Scheme 2:**
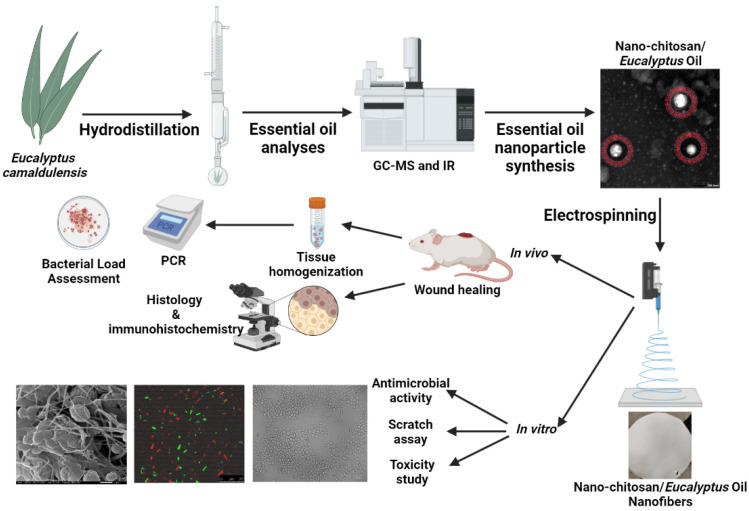
Schematic representation of the study plan.

Executions occurred after 5, 10, and 15 days following wound treatment, and the wound area was gently cleansed with 70% ethanol and excised. Skin tissue was homogenized in 1 mL of phosphate buffer (PBS) under sterile conditions. After tissue homogenization (Scheme 2), it was serially diluted with PBS before being plated on blood agar and incubated for 24 h at 37 °C. The number of viable cells (CFU/gram tissue) was calculated using Equation (4) [21,26]:

(4) $$CFU/gram\;of\;tissue=plate\;count\left(\frac{1}{dilution}\right)\times \left(\frac{10}{weight\;of\;tissue}\right)$$

#### 2.8.3. Histological Studies

Skin was dissected from each rat in the tested groups and was histologically analyzed. Histological analysis of tissue samples was conducted by fixing them in 10% formalin, which allowed the deep penetration of the fixative. The fixed samples were dehydrated using ethanol, then Paraplast was used as the embed material. Masson’s trichrome was used to stain the tissue sections (5 µm thickness).

#### 2.8.4. Immunohistochemistry (IHC)

IHC was conducted on about 4 µm thick paraffin sections of positively charged slides. Deparaffinization, rehydration and epitope exposure were evaluated with a 0.01 M citrate buffer (pH 6.0) for 10 min. The endogenous peroxidase activity was blocked, then the sections were rinsed with phosphate-buffered saline (PBS) and incubated for one hour at room temperature with the primary antibodies directed against the monoclonal mouse anti-human CD31 antibody (Dako corporation product clone: JC70A species, IgG1, kappa). A standard avidin-biotin-peroxidase technique was employed using diaminobenzidine (DAB, 5 min incubation) for visualization and hematoxylin for counterstaining (30 s). Appropriate negative controls, consisting of histologic sections without the addition of primary antibody, were prepared [38].

#### 2.8.5. RNA Extraction and RT-PCR Analysis

In a trial conducted to investigate the expression level of TGF-β1, type I collagen and type III collagen genes at each time point, a real-time polymerase chain reaction (RT-PCR) analysis was performed. The tested samples were homogenized, and the total RNA was extracted using the Wizol™ reagent (Wizbiosolutions, Gyeonggi-do, Republic of Korea) according to the product manual. The total RNA concentration and purity (OD260/280) were assessed using a NanoDrop spectrophotometer (Thermo Scientific, Waltham, MA, USA). Via quantitative PCR (qPCR), single-strand cDNA was synthesized from the obtained RNA using a WizScript™ RT master (Wizbiosolutions, Gyeonggi-do, Republic of Korea). The primer sequences for the target genes were listed in Table 1 [2]. All the data were the means of the three trials. The expression levels of each target gene were normalized with the 18S ribosomal gene as a housekeeping gene, and relative gene expression was calculated via the 2^−ΔΔCt^ method.

### 2.9. Statistical Analyses

All the obtained data are the means of three trials. All data were analyzed using SPSS 17.0 software (IBM, NY, USA) and expressed as the mean ± SD. One-way analysis of variance (ANOVA) and Student–Newman–Kells tests were used to analyze the differences among groups. At *p* < 0.05, the difference was deemed statistically significant. All graphs were drawn using Origin 9.1 software (Origin Lab., Northampton, MA, USA).

## 3. Results and Discussion

### 3.1. Antimicrobial Activity of Essential Oils

Data in Table 2 revealed that the inhibition zone (IZ) diameter of *Eucalyptus* oil ranged from 10.0 to 15.0 mm against the pathogens tested. The highest IZ diameter (15.3 mm) was noticed against *S. aureus*. *Eucalyptus* oil and exhibited bactericidal activities with average MIC and MBC values ranging from 16.0 to 1250.0 μg/mL and from 256.0 to 1250.0 μg/mL, respectively, against the tested pathogens. However, the lowest MIC and MBC values (16.0 and 256.0 μg/mL, respectively) were detected against the *S. aureus* strain (Table 2). Similar results were reported by Mumu and Hossain [39], who mentioned that EO showed maximum (100%) inhibition activity against *S. aureus*, *P. vulgaris* and *A. hydrophila.* On the other hand, Aldoghaim et al. [40] stated that *Eucalyptus* oils showed variable antimicrobial activity against the tested microorganisms and *A. baumannii* was the most sensitive microorganism, followed by *S. enterica Typhimurium* and *E. coli*, while the MIC index proved the cidal effect of *Eucalyptus* oil [40]. MIC values of *E. camaldulensis* ranged from 0.08 μg/mL to 0.22 μg/mL against *Bacillus cereus* and *Staphylococcus aureus*, respectively [41]. Variations in the reported antimicrobial activity of *Eucalyptus* oil may be attributed to the different compositions of the oils used in the experiments, as well as variability in the experimental settings, which might account for these contrasting findings. However, *E. camaldulensis* might be a significant resource for the development and formulation of antibacterial and antifungal medications due to its inhibitory properties against the growth of several pathogenic microbes.

### 3.2. Eucalyptus Oil Characterization Using GC-MS and FTIR

*Eucalyptus* oil was prepared and analyzed using GC-MS as shown in Figure S1. The α-Pinene dimer, camphene, homovanillic acid, eucalyptol (1,8-cineole), and á-Ocimene were identified by referring to the corresponding acquisition time. However, eucalyptol (monoterpenoid) had the highest percentage (36.49%) followed by homovanillic acid (35.06%) (Table 3). Salem et al. [42] investigated the chemical composition of *Eucalyptus* leaf essential oil from the *Eucalyptus camaldulensis* trees grown in northern Egypt and reported that the main constituents of the extracted oil were 1,8-cineole (21.75%), and β-pinene (20.51%). Celeiro et al. [43] detected the presence of homovanillic acid (39.53%) in *Eucalyptus camaldulensis* extracts. Moreover, it was reported that the highest prevalent constituents in the *Eucalyptus* leaf oil were p-cymene, eucalyptol (1,8-cineole), α-pinene and α-terpinol (42.1, 14.1, 12.7 and 10.7%, respectively) [44]. The aforementioned results proved that the extracted *Eucalyptus* leaf oil is a rich source of monoterpene hydrocarbons and oxygenated monoterpenes. On the other hand, FTIR analysis was used to assess the structural and functional groups of *Eucalyptus* oil. FTIR spectral details showed CH stretching in methyl and methylene groups (2965.1 and 2920.8 cm^−1^), CH asymmetric deformation (1464.5 cm^−1^), CH_3_ symmetrical deformation (1374.3–1305.4 cm^−1^), C–O–C symmetrical (1078.9 cm^−1^) and asymmetrical (1213.6 cm^−1^) stretching, and CH and C-O deformations (1052.7–763.8 cm^−1^) (Figure S2).

### 3.3. Antimicrobial Activity of the Prepared Nano-Chitosan/Eucalyptus Oil

Nano-chitosan, and nano-chitosan/*Eucalyptus* oil were synthesized, and their antimicrobial activities were assessed. Data in Table 4 reveal that nano-chitosan/*Eucalyptus* oil showed the highest antimicrobial activity against the tested pathogens and that the highest inhibition zone diameter was recorded against *S. aureus* (43.0 mm). Based on the obtained results, nano-chitosan/*Eucalyptus* oil was chosen for further studies. Darwish et al. [45] encapsulated *Eucalyptus staigeriana* essential oil in biopolymer matrices (consisting of aloe vera-coated dextran sulfate/chitosan nanoparticles) which inhibited bacterial growth by 47.27%. On the other hand, Sugumar et al. [46] formulated a *Eucalyptus* oil (*Eucalyptus globulus*)-impregnated chitosan nanoemulsion with a low droplet size and high stability with the antibacterial activity reaching a 15 mm inhibition zone diameter against *Staphylococcus aureus.*

### 3.4. Physico-Chemical Characterization of Nano-chitosan/Eucalyptus Oil

**Figure 1:**
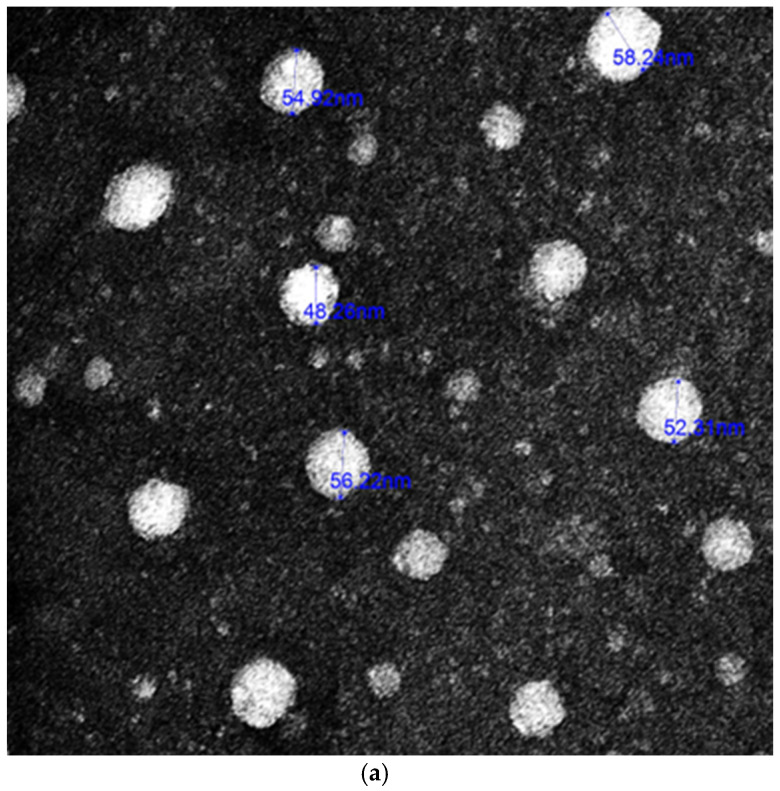

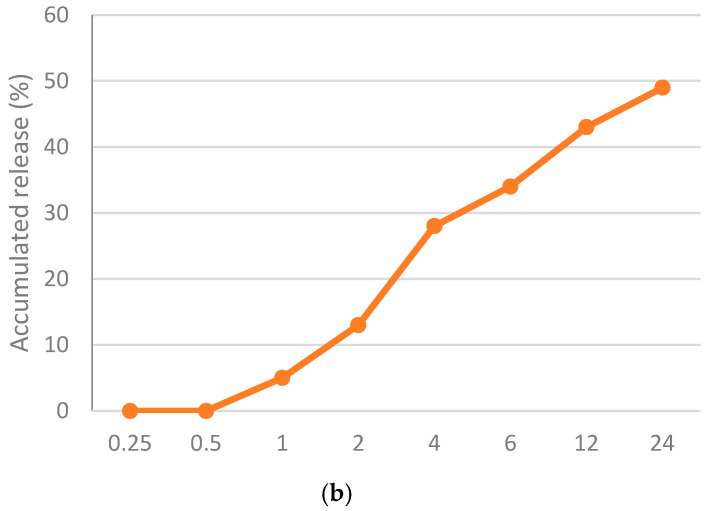
Physico-chemical characteristics of the prepared nanoparticles; (**a**) transmission electron micrograph of nano-chitosan/*Eucalyptus* oil and (**b**) in vitro release of *Eucalyptus* oil from the prepared nanoparticles.

The physico-chemical characteristics of the most potent nanoformula (Nano-chitosan/*Eucalyptus* oil) were assessed. TEM showed that the particle size of nano-chitosan/*Eucalyptus* oil ranged from 48.26 nm to 58.24 nm (Figure 1a), while the PDI and Zeta potential were 0.320 and 19.0 mV, respectively, which proved the stability and the relative homogeneity of the synthesized nanoparticles. The loading efficiency percentage (LE%) of the synthesized nanoparticles was 92.4%, which may explain the observed high antimicrobial effect of the synthesized nanoparticles. The release behavior of *Eucalyptus* oil from the prepared nanoparticles was assessed for 24 h in PBS. At the end of the experiment, only 49% of *Eucalyptus* oil was released from nano-chitosan/*Eucalyptus* oil (Figure 1b).

### 3.5. Morphological and Physical Characterizations of the Prepared Electrospun Nanofibers

**Figure 2:**
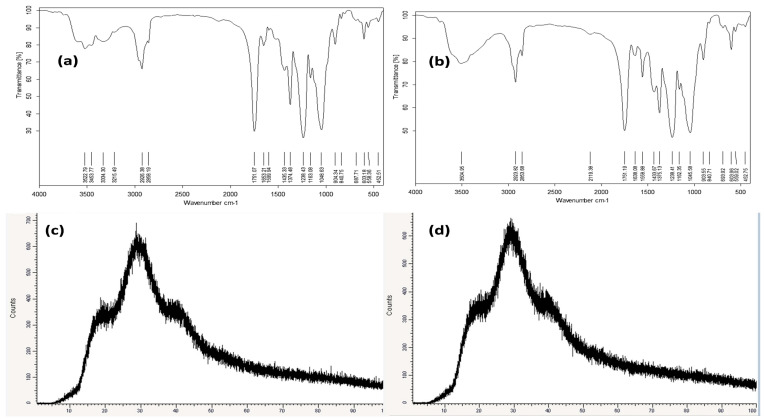
FTIR and XRD analyses of *Eucalyptus* oil/cellulose acetate nanofibers (**a**,**c**) and nano-chitosan/*Eucalyptus* oil/cellulose acetate nanofibers (**b**,**d**).

Nanofibrous membranes were prepared for both *Eucalyptus* oil and nano-chitosan/*Eucalyptus* oil-blended cellulose acetate followed by morphological and physical characterization assessments. According to the FTIR spectra of the *Eucalyptus* oil/cellulose acetate nanofiber (Figure 2a), the peaks at 3522.79–3334.49 cm^−1^ could be attributed to the O–H stretching vibration of the cellulosic matrix. However, 2926.38 cm^−1^ was attributed to CH– stretching, the peak at 1751.07 cm^−1^ was assigned to the C=O carbonyl group, and the peaks at 1163.08 cm^−1^ and 1048.63 cm^−1^ belonged to C–O– stretching, while the FTIR spectra of the nano-chitosan/*Eucalyptus* oil/cellulose acetate nanofiber (Figure 2b) were characterized by the presence of the functional groups characteristic of this nanofiber. What was noted was the appearance of broad absorption at 3504.95 cm^−1^ of the stretching vibration of the O–H groups. Moreover, the spectrum showed the existence of the absorption bands of two vibration modes of symmetrical vibration at 2923.82 cm^−1^ and of an asymmetrical one at 2853.68 cm^−1^ of the C-H groups. Moreover, the crystallinity of the *Eucalyptus* oil (Figure 2c) and nano-chitosan/*Eucalyptus* oil/cellulose acetate nanofibers (Figure 2d) was investigated using XRD. The XRD patterns of *Eucalyptus* oil/cellulose acetate and nano-chitosan/*Eucalyptus* oil/cellulose acetate nanofibers were similar with slight shifting in the nano-chitosan/*Eucalyptus* oil/cellulose acetate nanofiber pattern due to the change from *Eucalyptus* oil to nano-chitosan/*Eucalyptus* oil. In addition, their main diffraction peaks were located at 2θ = 17.5° and 28°, which corresponded to the (110) and (200) diffraction planes, respectively. Furthermore, the XRD analysis revealed that the chemical and mechanical methods had no effect on the crystal structure.

**Figure 3:**
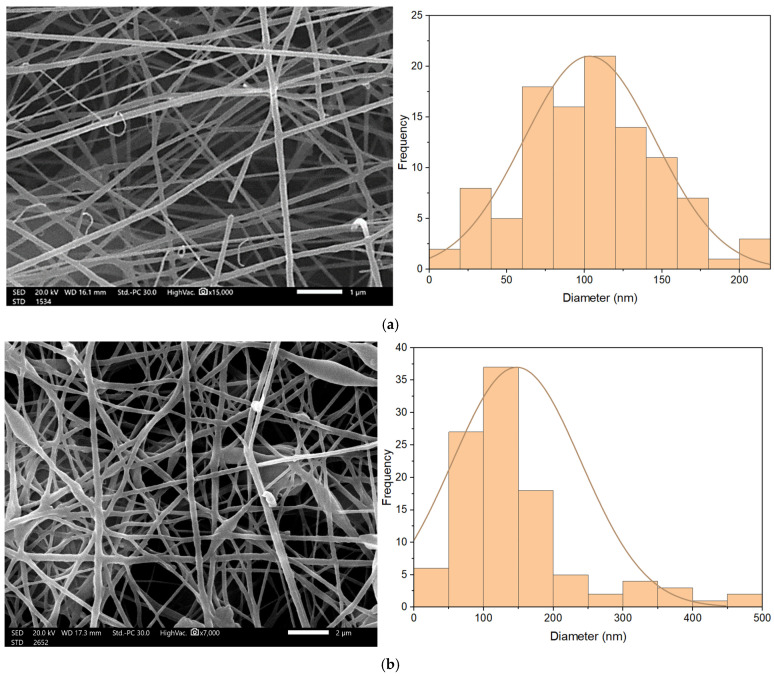

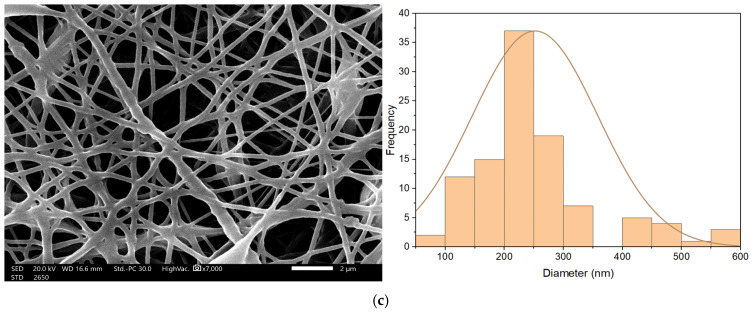
SEM of cellulose acetate nanofibers (**a**), *Eucalyptus* oil/cellulose acetate nanofibers (**b**) and nano-chitosan/*Eucalyptus* oil/cellulose acetate nanofibers (**c**).

**Figure 4:**
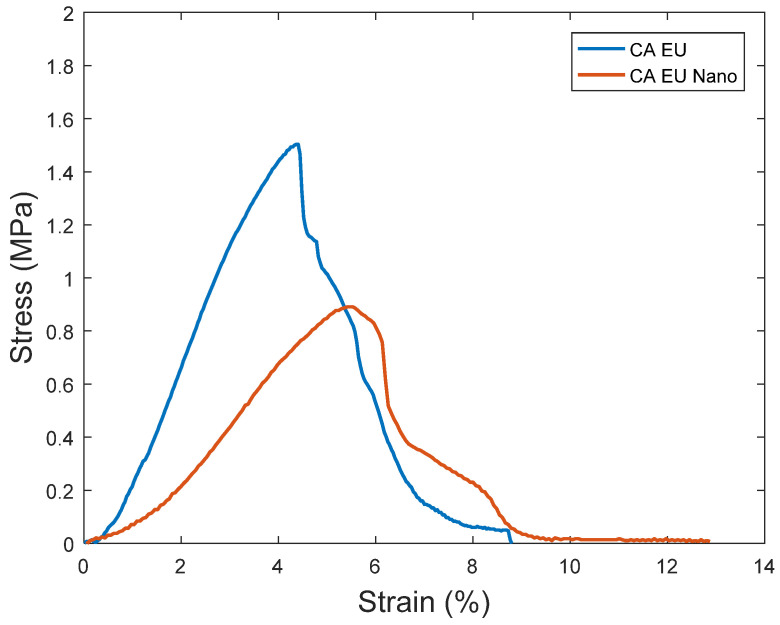
Stress–strain curves of the synthesized nanofibers. CA EU: *Eucalyptus* oil/cellulose acetate nanofibers; CA EU Nano: nano-chitosan/*Eucalyptus* oil/cellulose acetate nanofibers.

**Figure 5:**
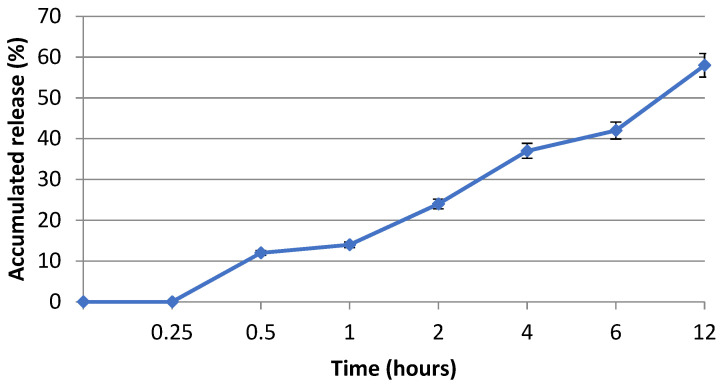
*Eucalyptus* oil release behavior of the fabricated nanofibers.

Nanofibrous membrane morphology was examined via scanning electron microscopy (SEM). The average fiber diameter of the nanofibers was obtained using Image-J software (2023 version). The SEM images revealed that the nano-chitosan/*Eucalyptus* oil/cellulose acetate nanofibrous membrane had a homogenous structure with a fine diameter of about 98.0 nm compared to *Eucalyptus* oil/cellulose acetate nanofibers and cellulose nanofibers which had a diameter of 115 and 180 nm, respectively. The addition of nanoparticles did not affect the fiber diameter as a result of the good dispersal, small particle size, and optimized spinning conditions (Figure 3). Figure 4 shows the stress–strain curves of the differently formed nanocomposites. One can notice that the composite of *Eucalyptus* oil/cellulose acetate showed the highest maximum elastic stress of up to 1.52 MPa, the maximum elastic strain of ~4%, and an elastic modulus of 35 MPa, where it had a plastic region beyond a 4% elongation strain and a breaking point of up to 8.8%. In the case of nano-chitosan/*Eucalyptus* oil/cellulose acetate nanofibers, both the elastic modulus and maximum elastic stress were reduced down to 18 MPa and 0.88 MPa, respectively. However, the added nanoparticles enhanced the maximum elastic strain by close to 6% with a maximum breaking point in the plastic region of a higher range compared to that of the undoped nanoparticle nanocomposite membrane. Moreover, the release behavior of nano-chitosan/*Eucalyptus* oil from the prepared nanofibers was evaluated in PBS for 24 h. A sharp release was observed during the first 6 h with a sustained release that lasted until the end of the experiment which reached 60% (Figure 5).

### 3.6. In Vitro Studies

#### 3.6.1. Antimicrobial Activity of the Prepared Nanofibers

**Figure 6:**
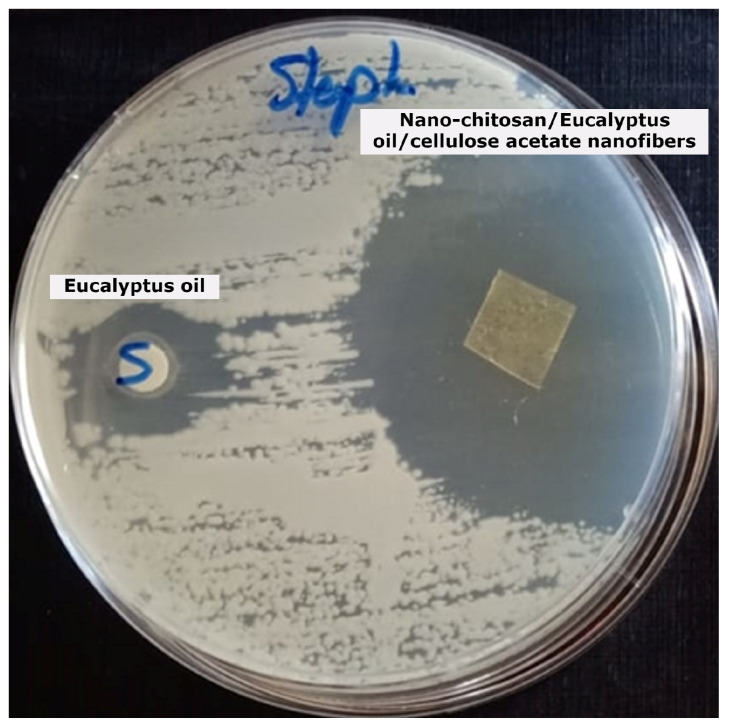
Antimicrobial effect evaluation via agar diffusion method.

**Figure 7:**
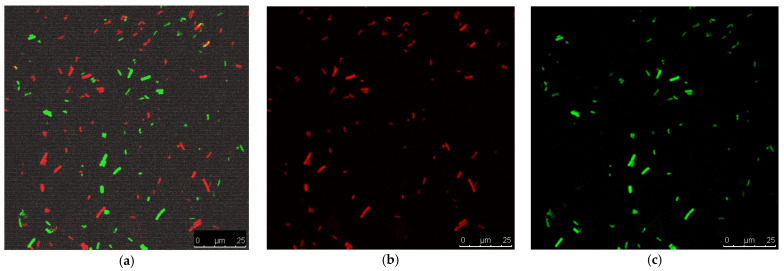
CLSM study of microbially treated cells; (**a**) merged image, (**b**) dead cells and (**c**) viable cells.

**Figure 8:**
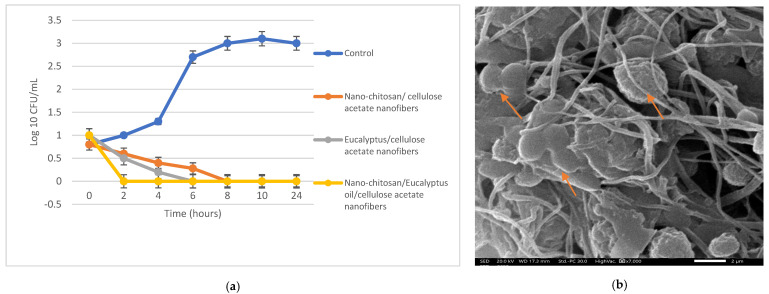
Antibacterial activity of Nano-chitosan/*Eucalyptus* oil/cellulose acetate; bacterial time–kill curve (**a**), SEM image of *S. aureus* treated cells (**b**) (arrows showing deformed bacterial cell).

The antibacterial activities of the prepared nanofibers were assessed through the ASTME 2149-01 standardized technique [30]. The growth reduction in % and agar diffusion effects after 24 h of incubation with the nano-chitosan/*Eucalyptus* oil/cellulose acetate nanofibers ranged from 65% to 87% and from 30.0 to 57.0 mm, respectively (Table 5). *S. aureus* treated with the nano-chitosan/*Eucalyptus* oil/cellulose acetate nanofiber showed the highest R% and highest agar diffusion results (Figure 6); therefore, it was selected for SEM and CLSM studies. The live (green)/dead (red) cell assay was assessed via confocal scanning laser microscopy (CLSM) (Figure 7a–c) to enumerate the microbial viable count which proved the growth reduction in % with increased red (dead) cells in comparison to green cells (viable). A SEM study was used to determine the mechanism of action of nano-chitosan/*Eucalyptus* oil/cellulose acetate nanofibers. Time–kill curve results revealed the potent impact of the fabricated nanofibers with complete bacterial cell eradication after 4 h (Figure 8a). Results revealed that *S. aureus* cells were adsorbed and adhered to the nano-chitosan/*Eucalyptus* oil/cellulose acetate nanofiber surface, which led to bacterial cell lysis and the release of the cell’s contents (Figure 8b). The possible potent antimicrobial mechanisms underlying the observed susceptibility can be explained by the differences in the outer membrane composition of the tested microorganisms [47]. Another possibility is the presence of certain oil components such as 1,8-cineole which can increase the permeability of the cytoplasmic membrane [40].

Behbahani et al. [48] studied the antimicrobial activities of carboxy methyl cellulose (CMC) films containing different *Eucalyptus camaldulensis* leaf extracts against some pathogens and reported that aqueous and alcoholic extracts were quite effective at a 2000 μg/mL concentration against *Streptococcus pyogenes* and *Staphylococcus epidermidis.*

#### 3.6.2. Evaluation of the Cytotoxic Effect of the Prepared Nano-Chitosan/*Eucalyptus* Oil/Cellulose Acetate Nanofibers

**Figure 9:**
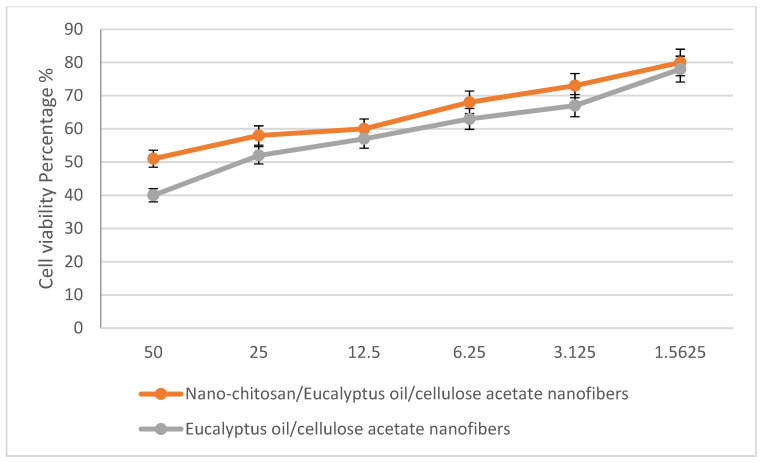
The percentage of normal melanocyte cell viability affected by nano-chitosan/*Eucalyptus* oil/cellulose acetate nanofibers.

In a trial studying the in vitro cytotoxic effect of the biosynthesized nano-chitosan/*Eucalyptus* oil/cellulose acetate nanofibers, cell proliferation using the human normal melanocyte cell line (HFB4 cells) was investigated. It was found that with 12.5 mg/mL of nano-chitosan/*Eucalyptus* oil/cellulose acetate nanofibers, the melanocyte cell viability was 60.0%, while with 1.5 mg/mL of nano-chitosan/*Eucalyptus* oil/cellulose acetate nanofibers and *Eucalyptus* oil/cellulose acetate nanofibers, the cell viability was 80.0 and 78%, respectively (Figure 9). Juergens [49] reported that *Eucalyptus* oil and 1,8-cineole have substantial promise as therapeutic agents against respiratory tract illnesses. The toxicity of *Eucalyptus* oil has been investigated in vitro as well as in vivo, and the latter studies revealed that 1,8-cineole showed no harmful effect [50]. Furthermore, human volunteer studies showed that (when used appropriately) the oil showed low allergenicity and toxicity [50]. More clinical research will be needed to identify the antibacterial properties of *Eucalyptus* oil to demonstrate its applicability as a therapeutic agent [39].

#### 3.6.3. In Vitro Scratch Assay of the Prepared Nanofibers

**Figure 10:**
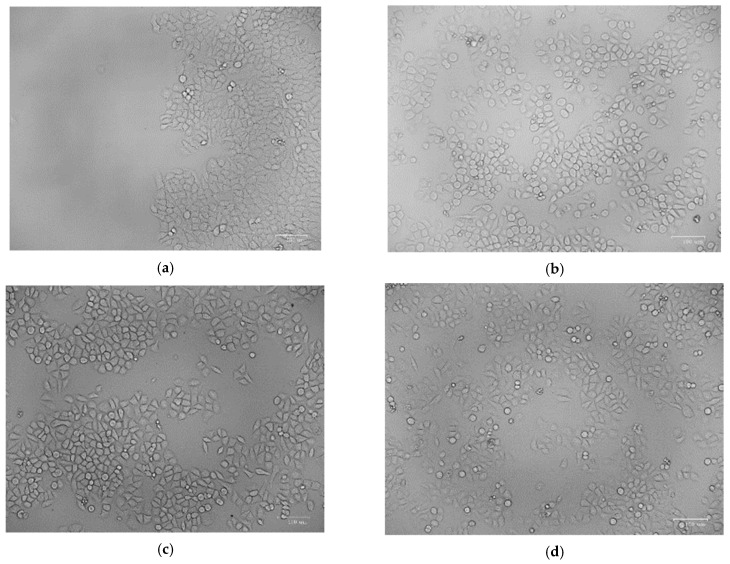
Analysis of cell migration using in vitro scratch assay; control (zero time) (**a**), control (24 h) (**b**), *Eucalyptus* oil nanofibers (24 h) (**c**) and nano-chitosan/*Eucalyptus* oil nanofibers (24 h) (**d**).

**Figure 11:**
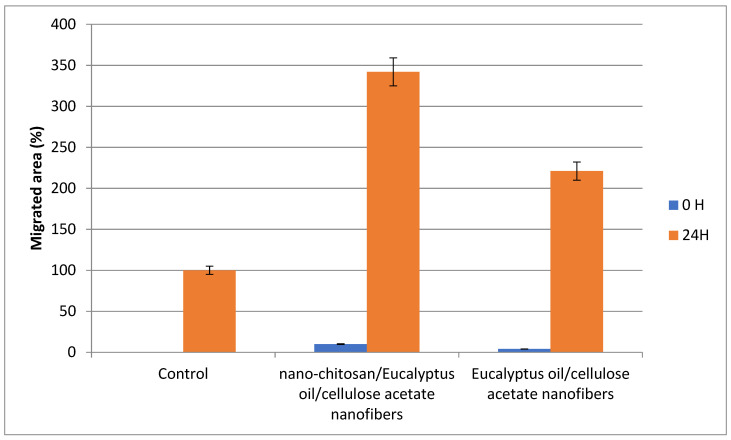
Scratch assay assessment of the migrated area % after 24 h.

The percentage of proliferative cells in the scratch borders or in peripheral areas of the monolayer was measured. After 24 h of incubation, the gap size of the tested groups decreased, with the maximum decrease was shown when the cells were treated with Nano-chitosan/*Eucalyptus* oil/cellulose acetate nanofibers, representing the significant migration of the cells toward the scratch (Figure 10 and Figure 11).

### 3.7. In Vivo Studies

#### 3.7.1. Morphological Study

**Figure 12:**
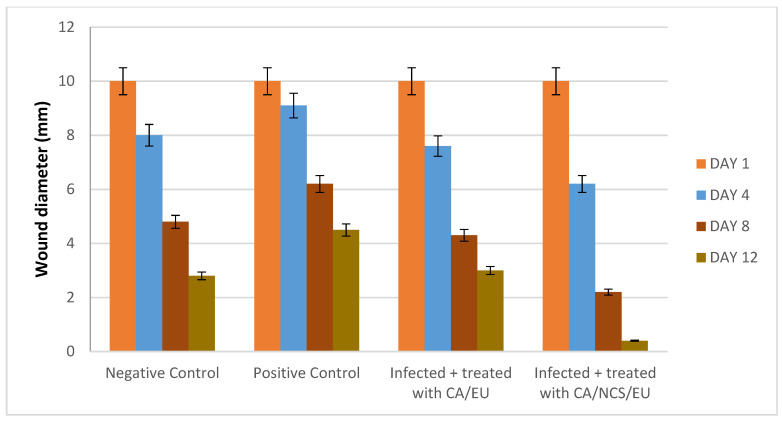
Wound healing diameter affected by time intervals. CA/EU: *Eucalyptus* oil/cellulose acetate nanofibers; CA/NCS/EU: nano-chitosan/*Eucalyptus* oil/cellulose acetate nanofibers.

Wound contraction in infected rats showed a gradual reduction of the wound area in the Nano-chitosan/*Eucalyptus* oil/cellulose acetate nanofibers compared to the control groups. Day 12 post-injury after treatment with Nano-chitosan/*Eucalyptus* oil/cellulose acetate nanofibers revealed 100% contraction of the wound area (Figure 12 and Figure S4).

#### 3.7.2. Bacterial Load Assessment

**Figure 13:**
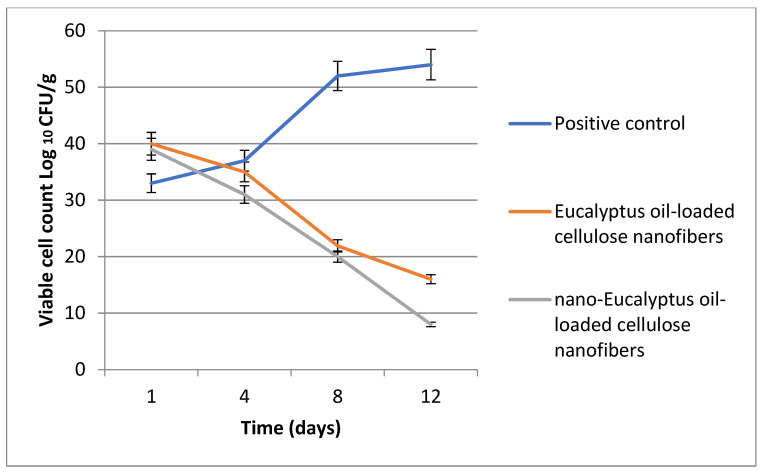
Bacterial cell load for the treated *S. aureus* wound infections.

At the beginning of the experiment, the rats received an intradermal injection containing 1.0 × 10^7^ CFU/mL of *Staphylococcus aureus*. Furthermore, at a specific time interval, the viable *S. aureus* cells were counted (CFU/g tissue) and the bacterial reduction in the infected skin wound post-treatment was determined for each group. Data in Figure 13 show a significant reduction in the bacterial cell count among all the treatments in relation to that of the control groups.

#### 3.7.3. Histopathological Investigations

##### First Interval

**Figure 14:**
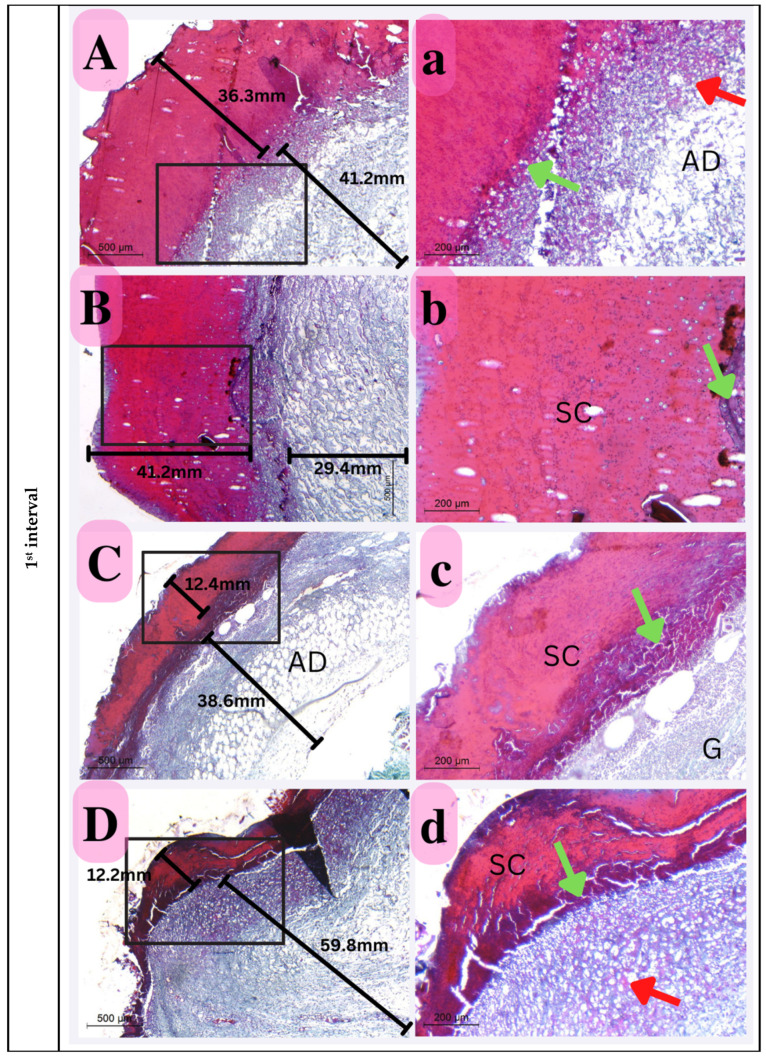

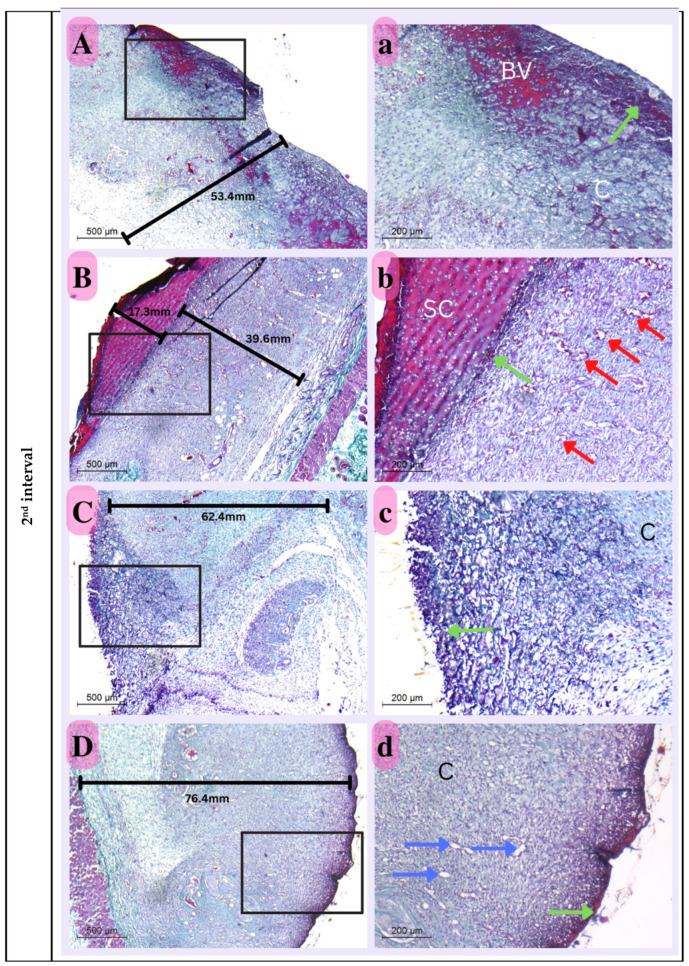

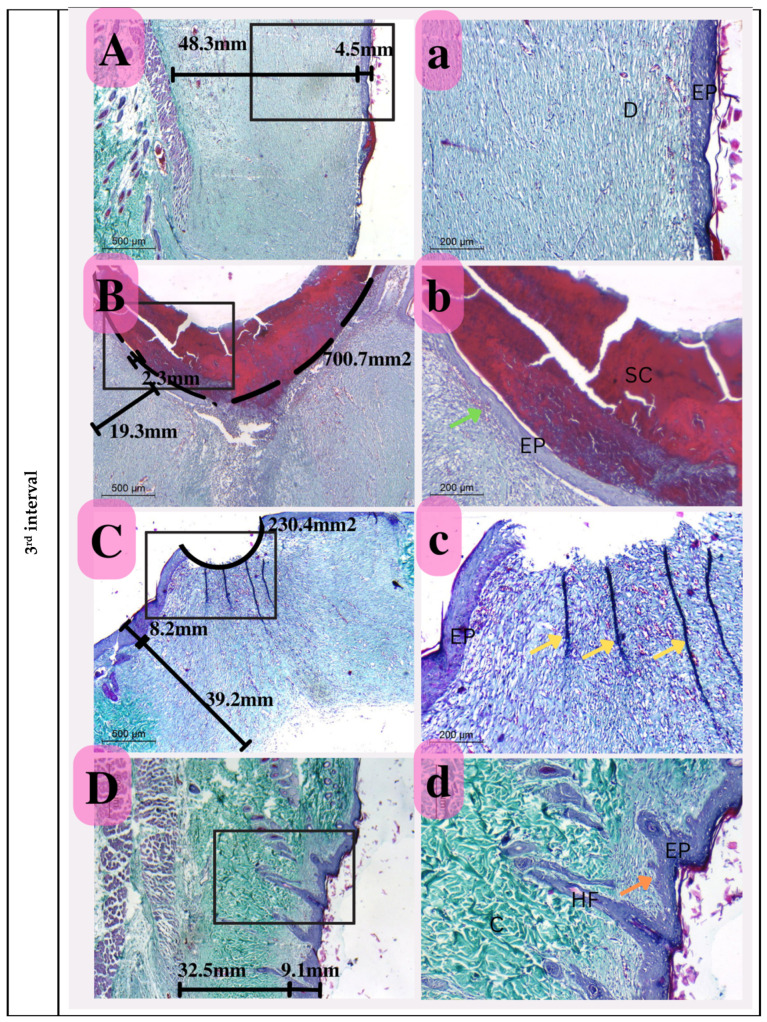
Photomicrograph of histological sections illustrating wound healing at the first interval stained with Masson’s trichrome (Capital letters ×500; small letters ×200). (**A**,**a**) represents the control group; (**B**,**b**) represents the positive control group; (**C**,**c**) represents the *Eucalyptus* oil/cellulose acetate nanofiber-treated group; (**D**,**d**) represents the nano-chitosan/*Eucalyptus* oil/cellulose acetate nanofiber-treated group. This photomicrograph shows inflammatory cells (green arrows), blood vessels (red arrows); adipose tissue (AD), scabs (SC), and granulation (G). Photomicrograph of histological sections illustrating wound healing at the second interval stained with Masson’s trichrome (×500; ×200). (**A**,**a**) represents the control group; (**B**,**b**) represents the positive control group; (**C**,**c**) represents the *Eucalyptus* oil/cellulose acetate nanofiber-treated group; (**D**,**d**) represents the nano-chitosan/*Eucalyptus* oil/cellulose acetate nanofiber-treated group. This photomicrograph shows inflammatory cells (green arrows), blood vessels (numerous red arrows), collagen (C), dilated blood vessels (blue arrows), congested blood vessels (BV), and a scab (SC). At the third interval stained with Masson’s trichrome (×500; ×200), (**A**,**a**) represents the control group; (**B**,**b**) represents the positive control group; (**C**,**c**) represents the *Eucalyptus* oil/cellulose acetate nanofiber-treated group; (**D**,**d**) represents the nano-chitosan/*Eucalyptus* oil/cellulose acetate nanofiber-treated group. This photomicrograph shows remnant inflammatory cells (green arrows), mature collagen (C), contractile lines (yellow arrows), a dermal papilla (orange arrow), a scab (SC), the epidermis (EP), and the dermis (D).

Skin sections indicated extensive epidermal damage in all the experimental groups, which was significant enough to separate them by thickness. Thus, compared to the control groups, the *Eucalyptus* oil/cellulose acetate nanofiber-treated groups showed much less epidermal layer degradation at this time period. Furthermore, the thickest damage scab was identified in the positive control, indicating that bacteria play a role in maximizing the damage epithelia. In addition to the above description, inflammatory cells dispersed underneath the damaged epidermis and visible angiogenesis promote recovery over time. The proportionate thickness of the dermal layer to the epidermis reveals a crucial element of the healing process; this was also visible in the nano-chitosan/*Eucalyptus* oil/cellulose acetate nanofiber-treated groups, with no significant difference between the two groups (Figure 14).

##### Second Interval

The thickness of the dermal layer was a significant criterion with which to assess the healing progress of the various experimental groups at this time period. The nano-chitosan/*Eucalyptus* oil/cellulose acetate nanofiber-treated group displayed the thickest dermal layer, which was significant in comparison to all other experimental groups, including the control group, suggesting the synergetic function of *Eucalyptus* oil in promoting dermal healing when mixed with nano-chitosan particles. Immature collagen was also detected in all experimental groups, with dispersed blood vessels in between to assist healing development. There was still widespread cellular infiltration, which played a role in epithelization (Figure 14).

##### Third Interval

This period was defined by considerable epithelization in the nano-chitosan/*Eucalyptus* oil/cellulose acetate nanofiber-treated group compared to that in the other groups and especially in the control group, indicating a strong synergistic function of nano-chitosan/*Eucalyptus* oil in enhancing epithelization rates. This group also showed a significant degree of ordered epithelization comparable to that of an intact epidermis with visible hair follicle development, which was also used to measure re-epithelization and identify unique healing for this group. The *Eucalyptus* oil/cellulose acetate nanofiber-treated group’s wound area did not close completely, indicating the significance of the prior description’s considerable synergetic impact. However, compared to the positive control, it showed a reduced wound area, showing the involvement of this oil in the healing process. Furthermore, the *Eucalyptus* oil/cellulose acetate nanofiber-treated group reflected certain contraction lines to enhance wound bed shrinking. In the positive control group, the presence of a big scab remaining connected to the wound region showed poor reepithelization as a consequence of infection (Figure 14).

##### Immunohistochemistry

**Figure 15:**
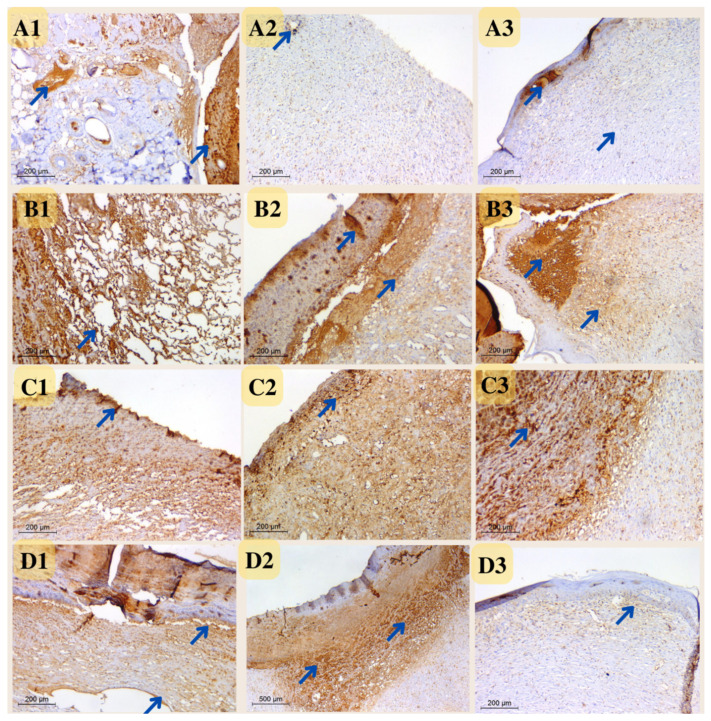
Photomicrograph of wound tissue sections stained with CD31 expression as angiogenesis factor in rat wounds on days 4 (coded number 1), 8 (coded number 2) and 12 (coded number 3) (×200). (**A1**–**A3**) control group; (**B1**–**B3**) positive control group; (**C1**–**C3**) *Eucalyptus* oil/cellulose acetate nanofiber-treated group; (**D1**–**D3**) nano-chitosan/*Eucalyptus* oil/cellulose acetate nanofiber-treated group. Arrows indicate the stained vesicles.

**Figure 16:**
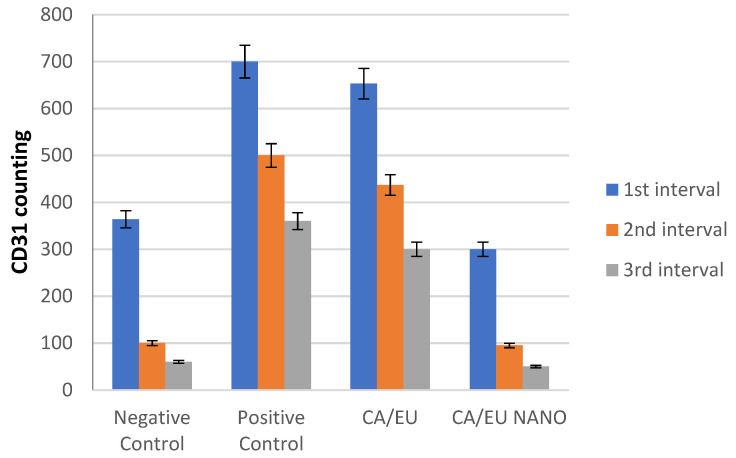
Immunohistochemistry counting of positive CD31 cells.

Microvessel density (MVD) was measured at five locations in three-time intervals using CD31-positive cells. The MVD difference across all experimental groups was difficult to discern during the first period. During the second interval, however, MVD was significant in both the nano-chitosan/*Eucalyptus* oil/cellulose acetate nanofiber and control groups. The MVD was not significant between the *Eucalyptus* oil/cellulose acetate nanofiber-treated group and the positive control group, indicating that *Eucalyptus* oil had no effect on the angiogenesis process at this time period. The third interval indicated that the nano-chitosan/*Eucalyptus* oil/cellulose acetate nanofiber-treated group had a much lower MVD than the control group did, indicating the synergistic impact of decreasing inflammation caused by the angiogenesis process. Based on the MVD identified in their tissue, those groups were often at the remodeling healing stage, as opposed to the other groups, which were delayed and still in the inflammatory healing stage (Figure 15 and Figure 16). CD31 is an important immunological factor that influences endothelial cell–cell adhesion, contact, and cellular transmigration and diapedesis, thus contributing to angiogenesis, vascular integrity maintenance, and wound healing at early stages [51,52].

##### TGF-β1, Collagen Type I and III Expression in the Wound Tissue

**Figure 17:**
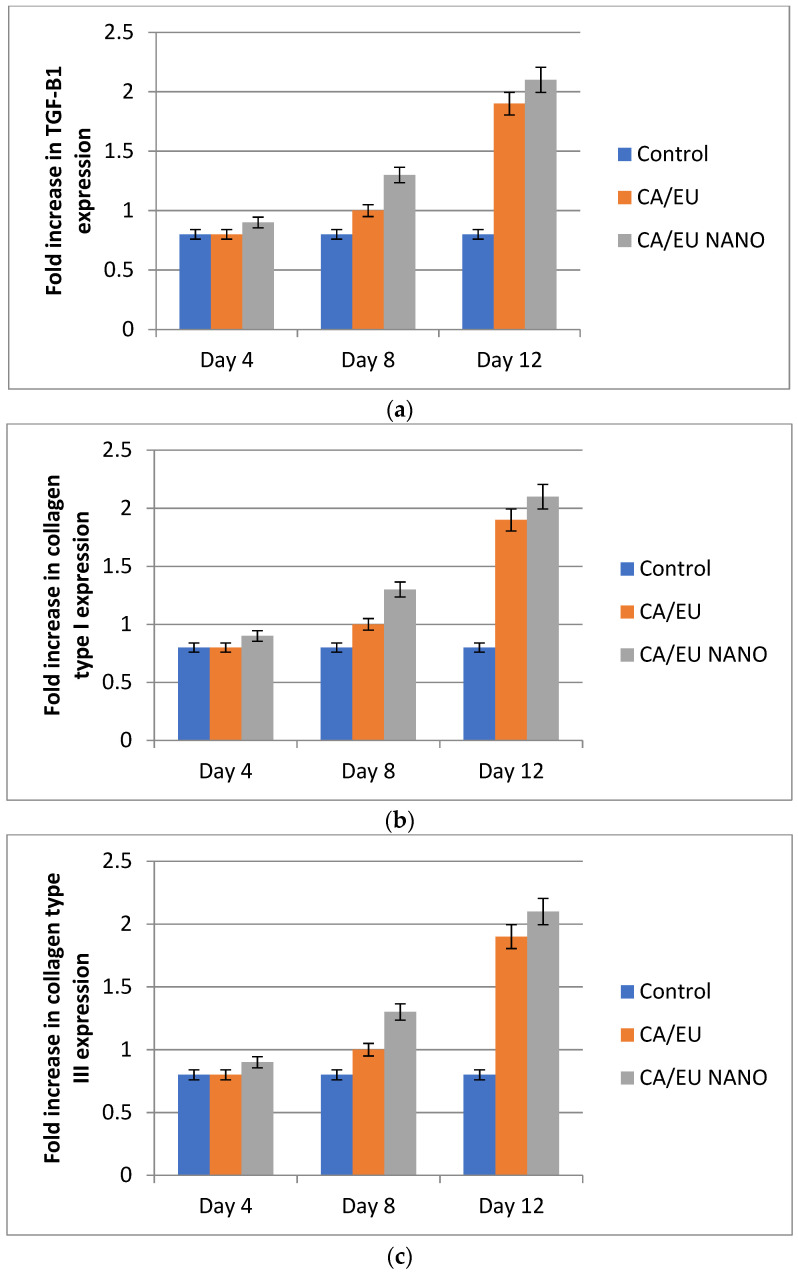
Fold increase of TGF-β1 (**a**), type I collagen (**b**), and type III collagen (**c**) gene expression. CA/EU: *Eucalyptus* oil/cellulose acetate nanofibers; CA/EU NANO: nano-chitosan/*Eucalyptus* oil/cellulose acetate nanofibers.

Further investigation of the fabricated nanofibers’ healing effect was carried out by evaluating mRNA expression levels of TGF-β1, type I, and type III collagen. RT-PCR results showed that the above-mentioned genes were up-regulated in all experimental groups compared to those of the untreated group on days 8 and 12 of treatment (Figure 17). The gene expression findings for TGF-1, type I, and type III collagen are in agreement with the histological findings of this research. The highest levels of TGF-1 and collagen were found in the cellulose/*Eucalyptus* oil and cellulose/nano-*Eucalyptus* oil nanofibers, which makes sense given the roles that these two factors play in the formation of granulation tissue and collagen, the main component of the extracellular matrix.

During the inflammatory phase of wound repair, local vasodilation and increased vascular leakage occur. Inflammatory cytokines and growth factors such as IL-1, PLGF, TNF-α, and TGF-β are released by activated macrophages. TGF-β controls fibroblast proliferation, collagen production, granulation tissue formation, and fibroblast differentiation into myofibroblasts in granulation tissue [53]. During the proliferative phase, wound cellularity increases because of the migration and proliferation of fibroblasts, endothelial cells, and keratinocytes, which controls angiogenesis, epithelialization, extracellular matrix (ECM) formation, and perfusion [54]. In the regeneration phase, the last phase of skin wound healing, cellular accumulation, angiogenesis, and collagen fiber deposition all decrease. Since collagen is a significant component of the extracellular matrix involved in wound healing, the accumulation and control of collagen strands are essential for wound healing. A recent study revealed that type III collagen levels increase during the onset of wound healing and are thereafter replaced by type I collagen levels. Wound healing is significantly affected by type III collagen, which is more active in platelet aggregation than type I collagen is [55]. Chitosan positively influences the healing process through the faster formation of granular tissue in the initial stages of healing [56].

## 4. Conclusions

Data of the present investigation concluded that the encapsulation of *Eucalyptus* oil in a nano-drug carrier greatly enhanced the antibacterial properties against the tested pathogens. A new nano-chitosan/*Eucalyptus* oil/cellulose acetate electrospun nanofiber was prepared and tested for in vitro and in vivo wound healing efficiencies. *Eucalyptus* oil was reported to have the highest inhibitory zone diameter, MIC, and MBC against *Staphylococcus aureus* (15.3 mm, 16.0, and 256 μg/mL, respectively). *Eucalyptus* oil encapsulated in chitosan nanoparticles greatly inhibited *S. aureus* growth with a 43 mm inhibition zone diameter. The biosynthesized nanoparticles had a 48.26 nm particle size, 25.5 mV zeta potential, and 0.415 PDI. Electrospinning nano-chitosan/*Eucalyptus* oil/cellulose acetate fabricated homogeneous nanofibers with a diameter of around 98.0 nm. Nano-chitosan/*Eucalyptus* oil/cellulose acetate nanofibers were safe and promoted wound healing by increasing TGF-β, type I, and type III collagen formation. This may pave the way to synthesize natural nanoparticles and formulate new polymeric electrospun wound dressings using *Eucalyptus* oil.

## Figures and Tables

**Table 1:** Primer sequences of the target genes.

Name	Primer Sequence
TGF-β1	F: ACTGATACGCCTGAGTGGCTGT
	R: CTCTGTGGAGCTGAAGCAGTAG
Type I collagen α2	F: CATGCCGTGACCTCAAGATG
	R: TCCATCGGTCATGCTCTCTC
Type III collagen α1	F: GAAGACGGCAAAGATGGGTC
	R: CATACCCCGTATCCCTGGAC
RNA 18S	F: GCAATTATTCCCCATGAACG
	R: GGCCTCACTAAACCATCCAA

**Table 2:** Antimicrobial activity of *Eucalyptus* oil against tested pathogens.

Tested Pathogens	Inhibition Zone Diameter (mm)	MIC (μg/mL)	MBC (μg/mL)	MIC Index
*K. pneumonia*	10.0 ± 1.0	256.0	1024.0	5.0
*P. aeruginosa*	10.0 ± 2.0	64.0	512.0	8.0
*P. vulgaris*	11.0 ± 2.0	64.0	512.0	8.0
*E. coli*	12.0 ± 4.0	64.0	512.0	8.0
*C. albicans*	11.0 ± 1.0	256.0	1024.0	5.0
*S. aureus*	15.0 ± 3.0	16.0	256.0	16.0
*A. baumannii*	11.0 ± 2.0	64.0	512.0	8.0

**Table 3:** GC-MS analysis of *Eucalyptus* oil.

RT	Compound	Area%
7.74	α-Pinene dimer	6.52
8.15	Camphene	0.08
8.85	α-Pinene	0.93
10.18	Homovanillic acid	35.06
10.35	Eucalyptol (1,8-cineole)	36.49
10.6	á-Ocimene	8.21

**Table 4:** Antimicrobial activity of the prepared nano-chitosan/*Eucalyptus* oil.

Tested Nanoformulae	Inhibition Zone Diameter (mm)			MIC (μg/mL)			MBC (μg/mL)		
	*P. vulgaris*	*C. albicans*	*S. aureus*	*P. vulgaris*	*C. albicans*	*S. aureus*	*P. vulgaris*	*C. albicans*	*S. aureus*
Nano-chitosan placebo	9.0 ± 1.0	9.0 ± 2.0	10.0 ± 4.0	512.0	512.0	256.0	1024.0	1024.0	512.0
Nano-chitosan/*Eucalyptus* oil	25.0 ± 3.0	21.0 ± 4.0	43.0 ± 6.0	32.0	64.0	4.0	256.0	256.0	64.0

**Table 5:** Antimicrobial activity of *Eucalyptus* and nano-chitosan/*Eucalyptus* oil/cellulose acetate nanofibers.

Nanofibers	R%			Agar Diffusion (mm)		
	*P. vulgaris*	*C. albicans*	*S. aureus*	*P. vulgaris*	*C. albicans*	*S. aureus*
Cellulose acetate nanofibers	0.0 ± 0.0	0.0 ± 0.0	0.0 ± 0.0	0.0 ± 0.0	0.0 ± 0.0	0.0 ± 0.0
Nano-chitosan/cellulose acetate nanofibers	10.0 ± 3.2	9.0 ± 1.0	12.0 ± 2.0	12.0 ± 5.0	10.0 ± 1.0	15.0 ± 2.0
*Eucalyptus*/cellulose acetate nanofibers	52.0 ± 9.4	49.0 ± 3.5	63.0 ± 4.0	15.0 ± 3.0	12.0 ± 3.0	20.0 ± 2.0
Nano-chitosan/*Eucalyptus* oil/cellulose acetate nanofibers	78.0 ± 5.0	65.0 ± 6.1	87.0 ± 2.4	42.0 ± 1.0	30.0 ± 1.0	57.0 ± 3.0

## Data Availability

The data of the current study will be available from the corresponding author on reasonable request.

## Supplementary Materials

The following supporting information can be downloaded at https://www.mdpi.com/article/10.3390/membranes13060604/s1. Figure S1: GC-MS chromatogram of *Eucalyptus oil.*; Figure S2: FTIR analysis of *Eucalyptus oil*.; Figure S3: Zeta potential (a) and zeta size (b) of the prepared oil nanoparticles; Figure S4: Morphological features of rats’ wounds.
