# Supplementary material for: Nano-Chitosan/Eucalyptus Oil/Cellulose Acetate Nanofibers: Manufacturing, Antibacterial and Wound Healing Activities
Source: Membranes (Basel). 2023 Jun 15;13(6):604. doi: 10.3390/membranes13060604 (PMC10301581; doi:10.3390/membranes13060604)
Supplement: Supplementary file 1 [file membranes-13-00604-s001.zip › membranes-2396976-supplementary.pdf]

Supplementary material

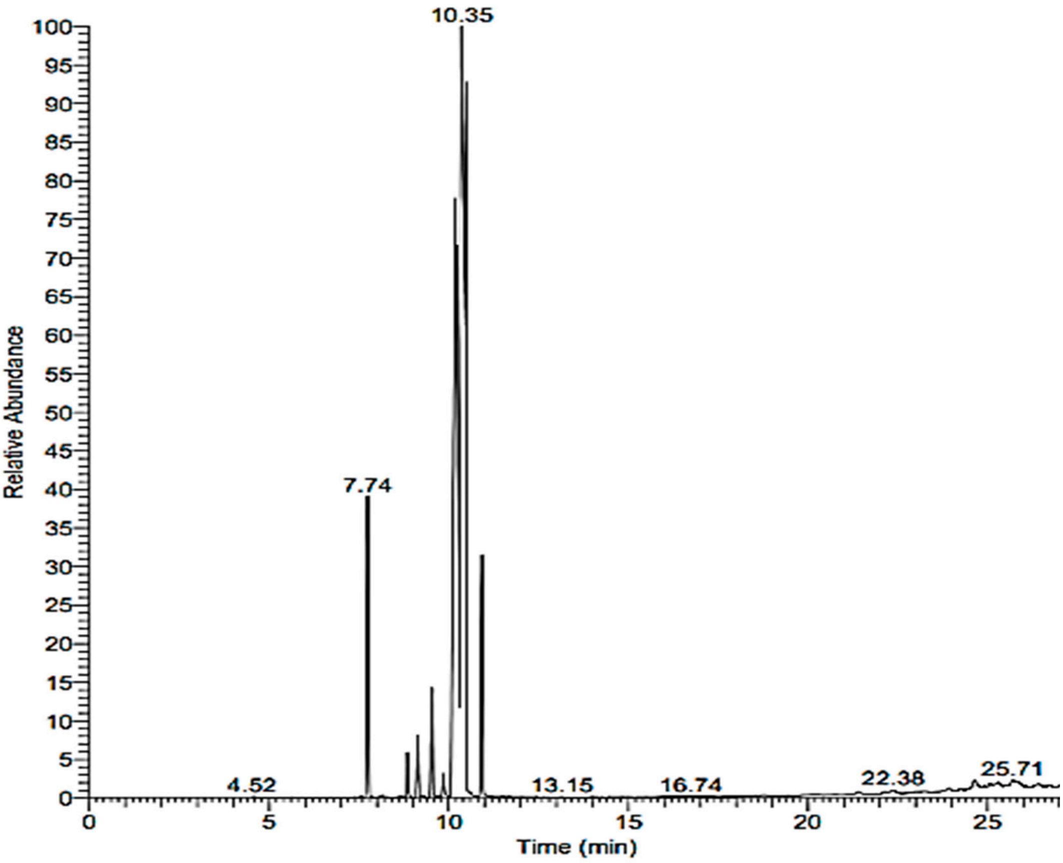

Figure S1. GC-MS chromatogram of *Eucalyptus* oil.

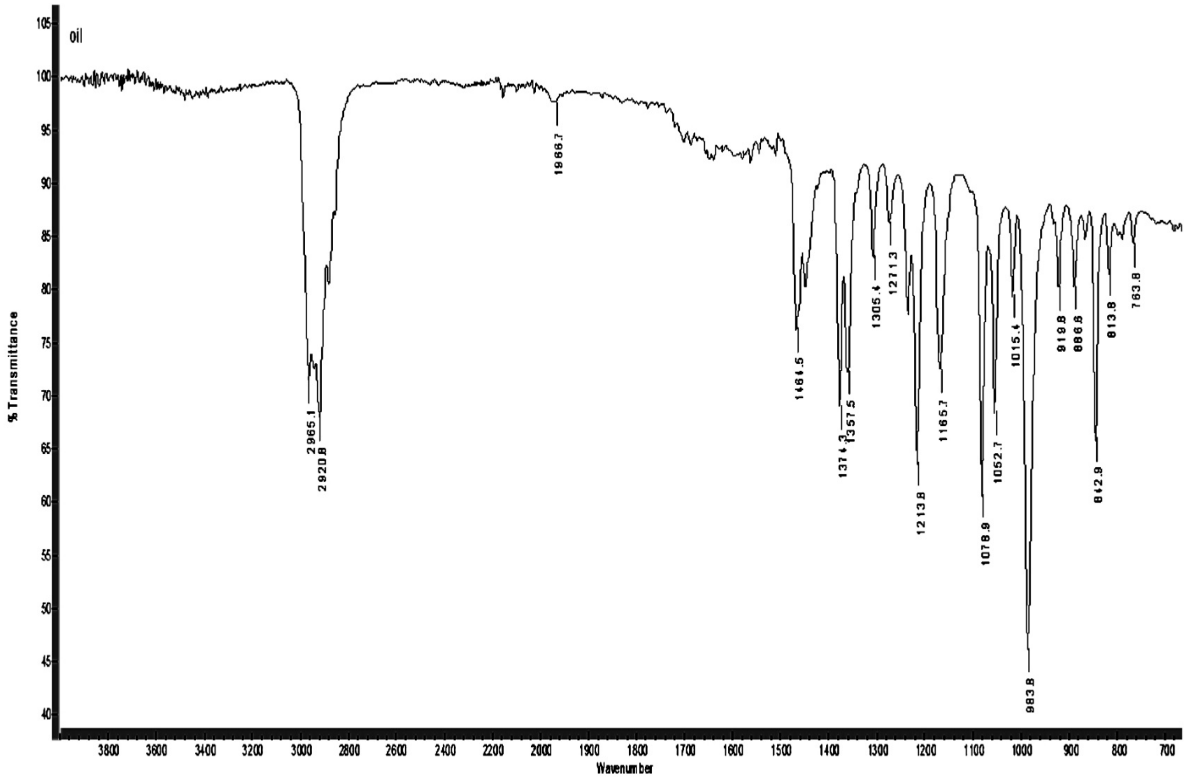

Figure S2. FTIR analysis of *Eucalyptus* oil.

			Mean (mV)	Area (%)	St Dev (mV)
Zeta Potential (mV):	19.0	Peak 1:	20.3	94.7	3.95
Zeta Deviation (mV):	5.12	Peak 2:	3.79	5.3	2.80
Conductivity (mS/cm):	0.185	Peak 3:	0.00	0.0	0.00
Result quality :	Good				

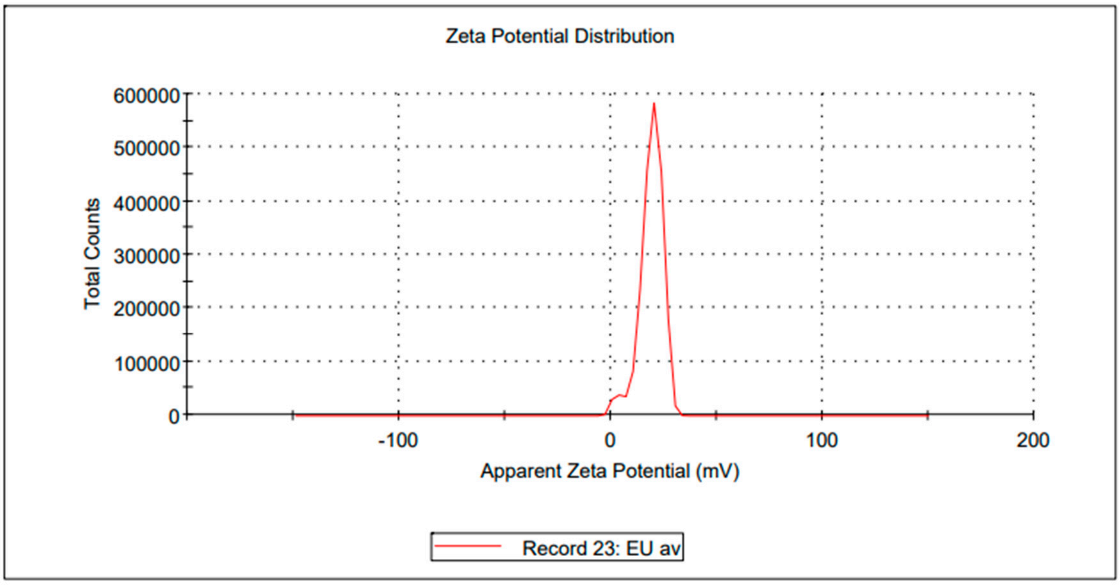

(a)

			Size (d.n...	% Intensity:	St Dev (d.n...
Z-Average (d.nm):	222.6	Peak 1:	277.9	95.6	176.6
PdI:	0.320	Peak 2:	4792	4.4	730.8
Intercept:	0.944	Peak 3:	0.000	0.0	0.000
Result quality	Good				

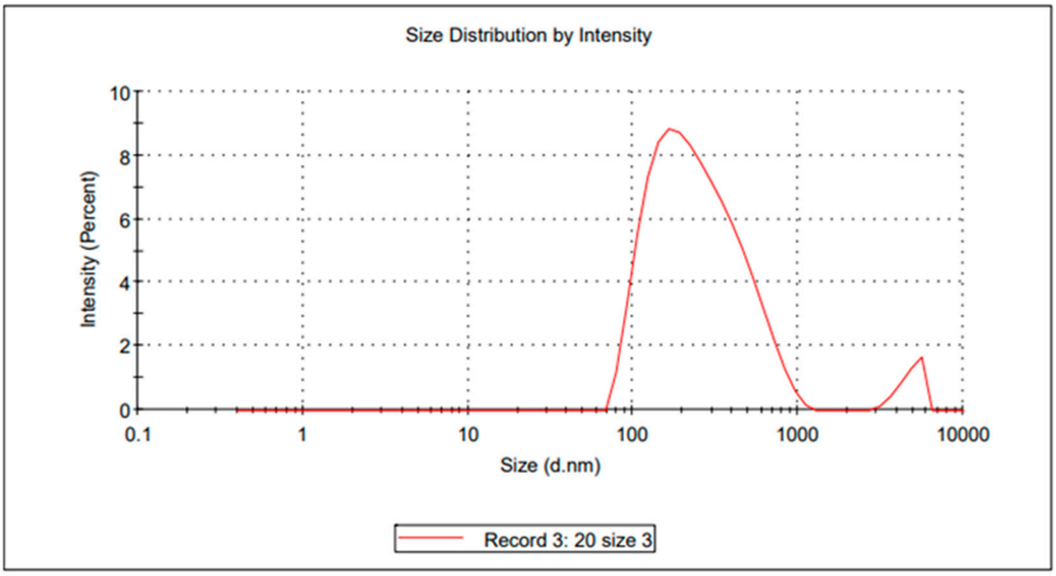

(b)

Figure S3. Zeta potential (a) and zeta size (b) of the prepared oil nanoparticles.

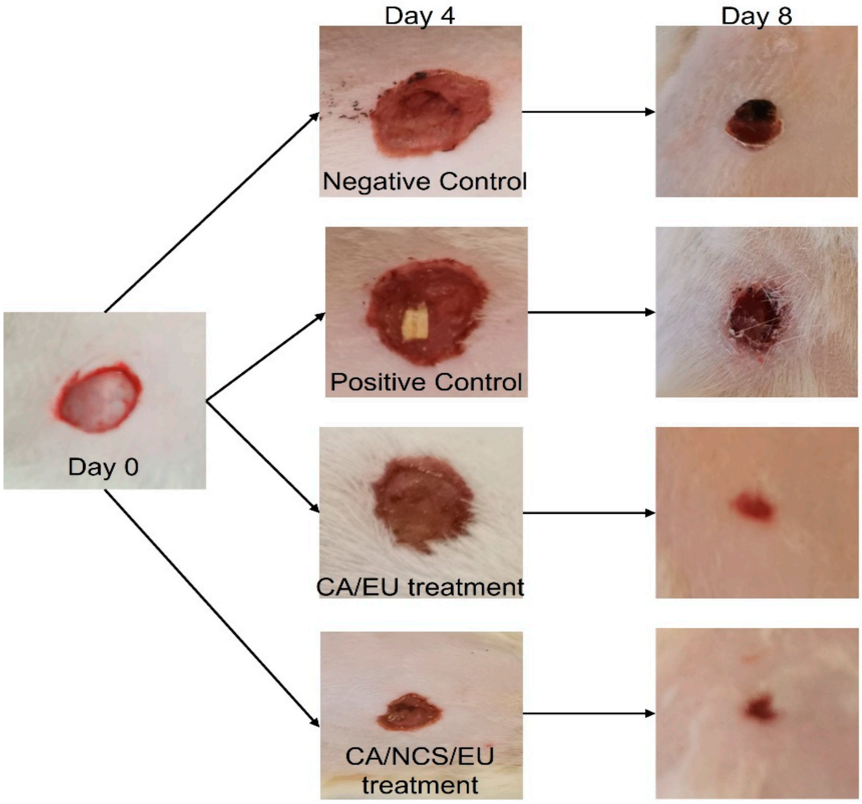

Figure S4. Morphological features of rats' wounds.
